# Molecular diagnosis and next generation gene sequencing in neuromuscular clinical practice

**DOI:** 10.1186/1471-2474-14-S2-O6

**Published:** 2013-05-29

**Authors:** Silvère van der Maarel

**Affiliations:** 1 Department of Human Genetics, Leiden University Medical Center, Leiden, The Netherlands

## 

Next generation sequencing (NGS) is revolutionizing the way we do research on genetic disorders. While earlier DNA sequencing was often the last step in disease gene discovery, nowadays it is becoming the starting point. Also, in the diagnostic setting, NGS is gaining momentum and is rapidly being implemented in laboratories around the world. With new developments trending toward more cost effective (label-free) technologies and higher throughput and fidelity, NGS instrumentation will undoubtedly become the gold standard. However, NGS in diagnostic and research settings will also impose new technical and computational challenges upon us, and raise ethical issues that will need to be addressed. Here I will discuss various NGS technologies and their specific characteristics and applications, with emphasis on translational research, bringing gene discovery into the diagnostic setting, and implications for the diagnosis of Pompe disease.

